# Mechanisms of Cultural Evolution in the Songs of Wild Bird Populations

**DOI:** 10.3389/fpsyg.2021.643343

**Published:** 2021-04-26

**Authors:** Heather Williams

**Affiliations:** Biology Department, Williams College, Williamstown, MA, United States

**Keywords:** birdsong, cultural evolution, learning biases, drift, conformity, sexual selection, directional selection, seeding

## Abstract

Young songbirds draw the source material for their learned songs from parents, peers, and unrelated adults, as well as from innovation. These learned songs are used for intraspecific communication, and have well-documented roles for such functions as territory maintenance and mate attraction. The songs of wild populations differ, forming local “dialects” that may shift over time, suggesting that cultural evolution is at work. Recent work has focused on the mechanisms responsible for the cultural evolution of bird songs within a population, including drift, learning biases (such as conformity and rare-form copying), and selection (including sexual selection). In many songs or song repertoires, variability is partitioned, with some songs or song segments being stable and consistent, while others vary within the population and across time, and still others undergo population-wide transitions over time. This review explores the different mechanisms that shape the cultural evolution of songs in wild populations, with specific reference to a long-term investigation of a single population of philopatric Savannah sparrows. Males learn a single four-segment song during their 1st year and sing the same song thereafter. Within this song, the buzz segment is a population marker, and may be stable for decades – variant forms occur but eventually disappear. In contrast, the middle segment is highly variable both within the population and over time; changes in relative prevalence of different forms may be due to cultural drift or a rare-form learning bias. Within the introductory segment, a high note cluster was replaced by a click train between 1982 and 2010, following an S-shaped trajectory characteristic of both selective sweeps in population genetics and the replacement of one form by another in human language. In the case of the Savannah sparrows, this replacement may have been due to sexual selection. In subsequent generations, the number of clicks within trains increased, a form of cultural directional selection. In contrast to the narrowing of a trait's range during directional selection in genetic systems, variation in the number of clicks in a train increased as the mean value shifted because improvisation during song learning allowed the range of the trait to expand. Thus, in the single short song of the Savannah sparrow, at least four different mechanisms appear to contribute to three different types of cultural evolutionary outcomes. In the future, it will be import to explore the conditions that favor the application of specific (and perhaps conditional) learning rules, and studies such as the ongoing song seeding experiment in the Kent Island Savannah sparrow population will help in understanding the mechanisms that promote or repress changes in a population's song.

## Introduction

The long history of studies of vocal learning in songbirds, whose songs vary widely in their characteristics across the 4,000+ known species, makes them ideal subjects for studying animal cultural evolution. That songbirds learn their songs was first demonstrated by William Thorpe, who raised birds in the laboratory, and, using the sound spectrograph, found that young birds accurately copied songs played to them during development (Thorpe, 1954). These observations have led to decades' worth of studies of captive birds, primarily canaries *(Serinus canaria)* and zebra finches *(Taeniopygia guttata)*, defining the factors that influence the timing and content of song learning. One major axis of variation in vocal learning across species, fortunately represented by the two main laboratory study organisms, is the timing of song learning: some species, including zebra finches, are called “critical period learners” or “age-limited learners,” and memorize a song model early in their lifetime, producing a crystallized version of that song when they first breed, and singing that same song for the rest of their lives (Immelmann, 1969). Other species are “open-ended learners”; canaries add some new material to their songs each breeding season, so that a bird's song changes from year to year (Nottebohm and Nottebohm, 1978). Still other species, such as mockingbirds *(Mimus polyglottus)*, are mimics, and can reproduce sounds they hear almost immediately (Kelley et al., 2008). Critical period learners preferentially memorize songs with species-specific acoustic characteristics during an early critical phase of development, demonstrating that birds have genetically determined species-specific sensory predispositions (Marler and Peters, 1988). Physical constraints within the vocal-motor system also play a role in defining what sounds can be learned and reproduced in a song; for example, fast trills of syllables with wide frequency transitions may be difficult to sing (Podos, 1996). Live tutors or even crude models that deliver songs when the young bird interacts with them result in more extensive and accurate learning than a model that is simply the sound of a song, highlighting the importance of social interactions (Baptista and Petrinovich, 1984; Adret, 1993). Although, for obvious reasons of stimulus control, experimental studies have primarily taken place in the lab, there is a notable exception in the form of a field study of learning in Savannah sparrows *(Passerculus sandwichensis*), a migratory species that breeds in North America, learns its song during a critical period, and returns to breed in its natal area. In this study, young males learned distinctive “foreign” songs from speakers placed in their natal area (Mennill et al., 2018). The learning of foreign songs replicated aspects of previous studies, including the timing of song learning.

**Figure 1:**
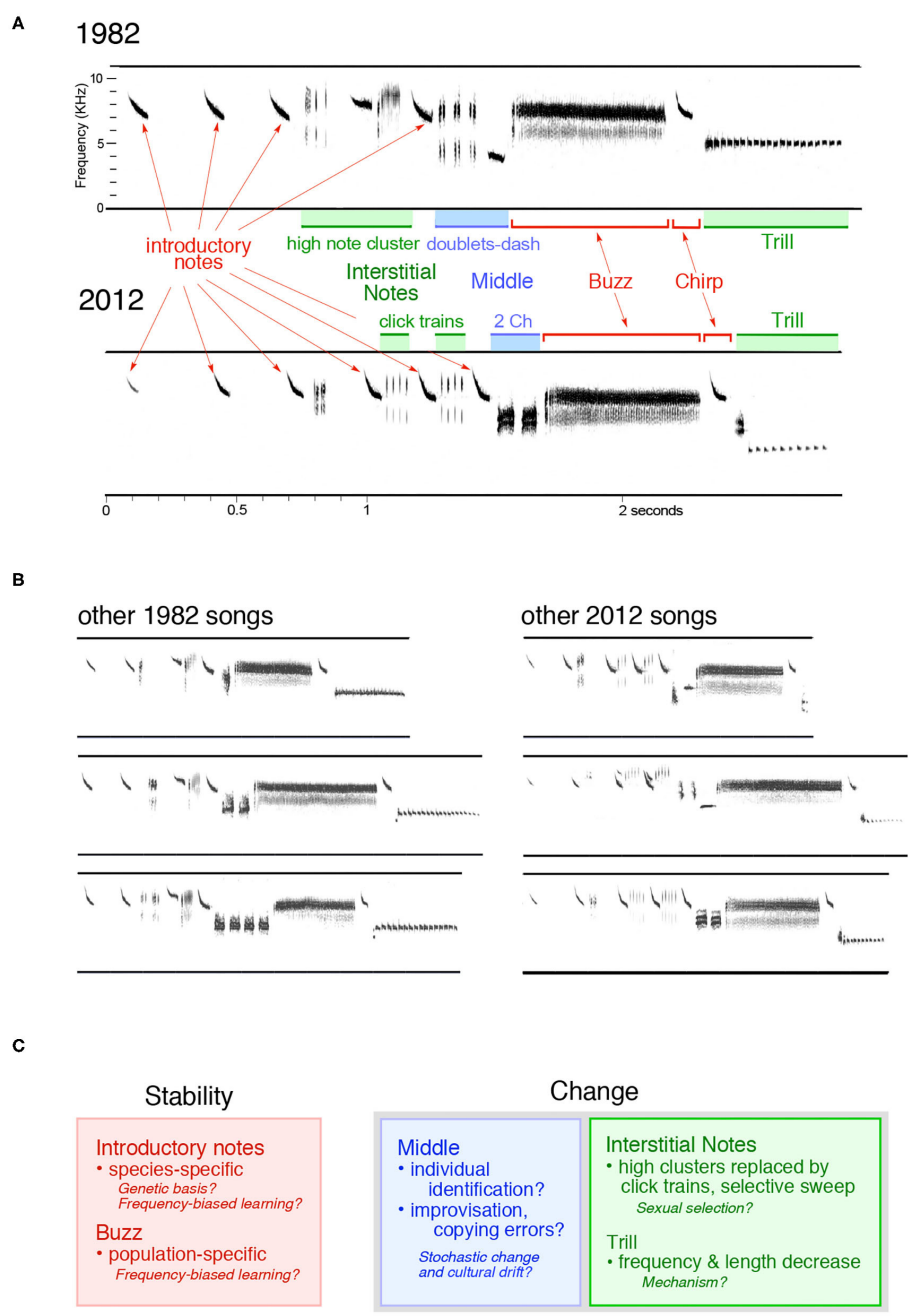
Cultural evolution in a Savannah sparrow population. **(A)** The two songs shown here, recorded 30 years apart, include the most common types of interstitial notes and middle notes sung in their respective years. Although some individual variation exists, introductory notes, buzzes and chirps (which match the introductory notes in this population) had stable acoustic characteristics, while interstitial notes, middle segments, and trills changed over time. **(B)** Additional examples of songs sung in 1982 and 2012 (initial introductory notes were omitted from some songs shown here). **(C)** The direction of song changes and possible explanations are summarized; no single mechanism can account for the cultural evolution observed in the songs of this population.

Within the limits of (a) critical period learning, which fixes a male's song in its crystallized form after his first breeding season, (b) genetically encoded predispositions, and (c) physical and physiological constraints, song learning in migratory songbirds such as the Savannah sparrow has considerable degrees of freedom^1^. Males learn from their social fathers (which may or may not be their genetic fathers), from natal year neighbors, and from neighbors early in their first breeding season (Wheelwright et al., 2008). Immigrants may sing songs that differ substantially from those of the local population, and improvisation and innovation by 1st-year birds may extend the range of variation of existing song types or introduce novel song elements. Such variation, if it is passed on through vocal learning, results in cultural evolution: change in the songs of a wild population over time. As we learn more about the characteristics and trajectory of such changes, it becomes increasingly apparent that cultural evolution differs between species, and that, even within the relatively simple, short song of the Savannah sparrow, no single mechanism can explain the changes we observe (Williams et al., 2013; Figure 1). Comparing the modes of cultural evolution across species and teasing apart the mechanisms that yield different rates and types of change within a single species will provide valuable insights into how and why different songs and song segments change in different ways, as well as into the cognitive mechanisms that are used by learners to apply different rules about copying, variability, and stability to different portions of a single song.

## Evidence for Cultural Evolution in Wild Songbirds

Changes in the song traditions of a population can be documented in three ways, ranging from strong inference to direct observation. The first method records songs across a range of sites and observes a geographic pattern of differences in the learned songs, with local populations sharing a dialect that differs from those of more distant groups. These geographical patterns suggest that dialects form as a result of different paths of cultural evolution in different populations. The second method records songs from the same population at two time points years or decades apart and notes changes in the local dialect. Such studies have been valuable in describing patterns of change that suggest mechanisms for shaping songs or song segments. The third method uses comprehensive recordings of individuals from a single population over a period of many years, providing a fine-grained data set that allows direct observation and resolution of the time course of changes in the songs of a population. The most powerful version of this population-focused method records the songs of color-banded individuals, and so includes demographic information such as hatching date and location, parentage, and reproductive success. The mechanisms of cultural evolution suggested by all three types of observational studies can then be modeled or experimentally tested.

### Geographic Differences

Peter Marler was the first to observe and define distinct dialects within a continuous distribution of chaffinches *(Fringilla coelebs)* in England and the Azores (Marler, 1952), as well as in white-crowned sparrows *(Zonotrichia leucophrys)* in the U.S. (Marler and Tamura, 1962). Where such dialects must have arisen within subpopulations of descendants of a single founder population within recent history, as in the case of the introduction of House finches *(Haemorhous mexicanus)* to the northeastern U.S. (Mundinger, 1975) and chaffinches to New Zealand and nearby islands (Lynch et al., 1989), the most parsimonious explanation is that local song traditions form after colonization and thus differences in local song traditions represent cultural evolution – as do, by analogy, dialects more generally. Geographically fine-grained studies have noted that some parts of the song may vary between individuals within a single population, while others are similar within a local population but vary geographically (Lee et al., 2019).

### Sampling at Different Time Points

Studies that return to previously recorded populations after a period of years or decades can document changes over time in the local traditions. In one population of chaffinches recorded 18 years apart, 8 of the original 23 song types could be recognized, but the other 15 song types could not be matched to later songs (Ince et al., 1980). Savannah sparrows recorded at the same locations in California after a gap of 15 years also lost elements and changed and added others, but many of the dominant song types were recognizable and the terminal flourish remained largely unchanged (Bradley, 1994). House finches recorded at the same locations in 1975 and 2012 retained some syllables even as the population, and the population's syllable diversity, expanded (Ju et al., 2019). White-crowned sparrows recorded in the 1970s and then again between 1997 and 2004 in the Puget Sound area had different patterns of change in different parts of the song: at each location, the terminal trill was stable over time, while another segment of the song, the note complex, changed (Nelson et al., 2004). In 1st-year birds, improvisation was 2.3 times greater in note complexes than in terminal trills. Terminal trills were also more similar to those of tutors (in laboratory studies) and neighbors (in field studies); improvisation and copy fidelity thus varied systematically for different parts of the song, resulting in different rates of cultural evolution within a single learned vocalization.

### Long-Term Studies

**Figure 2:**
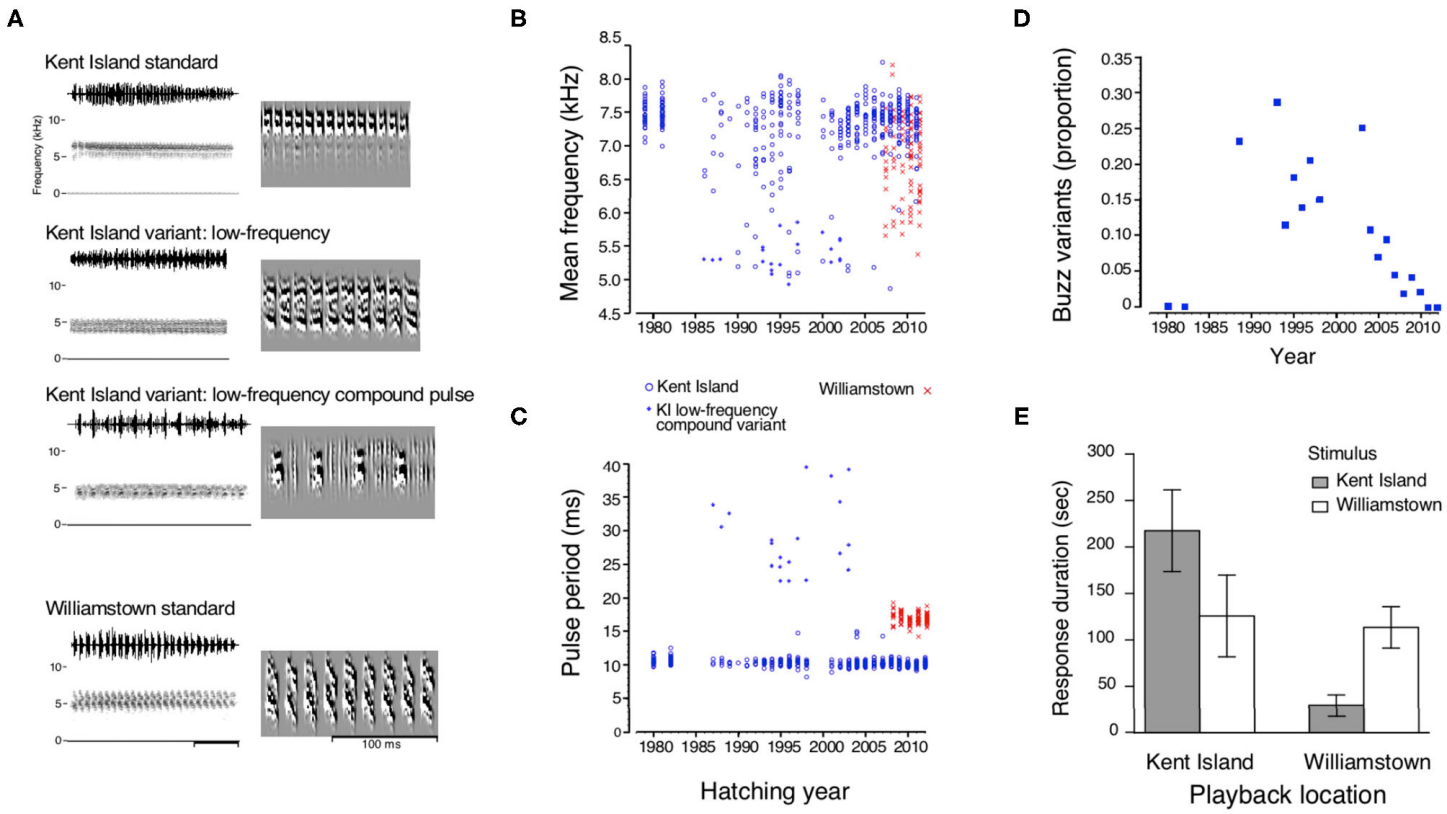
The population-specific buzz segment: maintenance of stability. **(A)** Sonograms of three forms of buzz sung by the Kent Island population and the single form sung by a 600 km distant population in Williamstown. Two low-frequency variants (differing in the duration and form of the constituent pulses) were sung by a total of 8.4% of the birds recorded on Kent Island between 1980 and 2012. **(B)** Distribution of mean frequencies and **(C)** pulse durations of buzzes sung by Kent Island and Williamstown birds. **(D)** The proportion of birds singing variant buzzes on Kent Island during years when recordings were made. **(E)** Males respond more aggressively, as measured by response duration, to the standard local buzz than to foreign buzzes (error bars represent 2 SEM) (After Williams et al., 2019).

The white-crowned sparrows of the Nelson group's Pacific Northwest field sites and Savannah sparrows at the Bowdoin Scientific station on Kent Island in New Brunswick have been systematically and comprehensively recorded over time. The Kent Island Savannah sparrows were first recorded in the early 1980s by Clara Dixon; a few recordings from 1988 and 1989 also exist, and extensive song recordings are available for the years from 1993 to 1998 and 2003 to 2019 (see Wheelwright et al., 2008). Because Nathaniel Wheelwright began a long-term population study that mapped territories and color-banded breeding adults as well as nestlings in 1987 in this philopatric population (Wheelwright and Mauck, 1998), an enterprise that was continued by Ryan Norris and his colleagues (Woodworth et al., 2017), we have extensive demographic data to go with the song recordings of identified males. Each male sings one song that does not change after crystallization early in the first breeding season (Wheelwright et al., 2008). As for the white-crowned sparrow, some portions of Savannah sparrow song, such as introductory notes and buzz, have been stable over time, while others, such as the interstitial notes and the middle section, have varied (Figure 1; Williams et al., 2013). This partitioning of variability between song segments in sparrows echoes differences in variability in the two song types of the chestnut-sided warbler *(Setophaga pennsylvanica)*. Across a period of 20 years, accented song types were stable, while unaccented songs varied (Byers B. E. et al., 2010). The stable, accented-ending songs are sung by unmated males seeking females and by mated males to their mates (Kroodsma et al., 1989), while males directing their songs at other males sing the variable, unaccented songs (Byers, 1996). The interplay of variation and stability in the two song types may promote their different functions. Savannah sparrow males respond more strongly to the local version of the stable, population-specific buzz segment (Williams et al., 2019; Figure 2E), and white-crowned sparrow males respond more strongly to the local version of the stable, population-specific trill segment (Nelson and Soha, 2004b). In contrast, female white-crowned sparrows approach all trill types (Nelson and Soha, 2004a). Like the song types of chestnut-sided warblers, different segments of these two sparrows' songs may be tailored to different audiences and serve different functions, and so lead to differences in rates and forms of cultural evolution.

## Mechanisms

By analogy to genetic evolution, cultural evolution occurs as a result of variation followed by differential transmission (Boyd and Richerson, 1985), resulting in a change in the composition of the learned vocalizations of a population of songbirds. Differential transmission may result from random processes, also known as cultural drift, or from selective processes. Cultural drift does not favor a particular result; what is retained and what is dropped by the population is determined stochastically, and so the result is not adaptive.

Darwinian selection may also take place during the transmission of learned song. Variation arises as a result of copying errors, improvisation, innovation, or immigration. Selection on the resulting song variants may be driven by (1) ecological factors such as environmental effects on sound transmission or salience, (2) sensory or motor biases that favor the learning or production of certain types of song content, (3) frequency-based learning biases that result in preferential learning of the most common song type (conformity) or in the copying of novel song forms (rare form advantage), and (4) prestige biases that favor imitation of certain models, such as dominant or successful individuals (Aplin, 2019; Whiten, 2019). Such learning biases require that the pupil observe and compare potential songs and song tutors. A young bird must distinguish between common and rare songs, and may also associate particular songs with knowledge of the singer's dominance status or mating success – which requires either direct or indirect observation of that success. Cultural evolution that is driven by selection based on social variables thus raises the question of whether a learner imitates the song or the singer.

In contrast to changes that result from drift, cultural evolution that is driven by selection should result in a song that is better able to carry information, or makes the singer better at acquiring territories or mates – in Darwinian terms, adaptation. Inferences about whether a song feature is adaptive can be made by tracking the success (in reproductive or cultural terms) of individuals singing a particular song feature, or by testing responses to that song in relation to its presumed adaptive function. Is a particular type of song transmitted better in an urban/forested/open environment? Do males or females respond differentially to songs with particular features?

For many years, the study of song variation in wild populations of a single species focused upon debates about the mechanisms that result in a pattern of dialects associated with specific geographic regions (Podos and Warren, 2007). More recently the focus has shifted to studying the mechanisms that result in change or stability of a population's song forms over a period of generations, the focus of this review. Some of these mechanisms are summarized in Table 1.

**Table 1:** Some mechanisms for the cultural evolution of song.

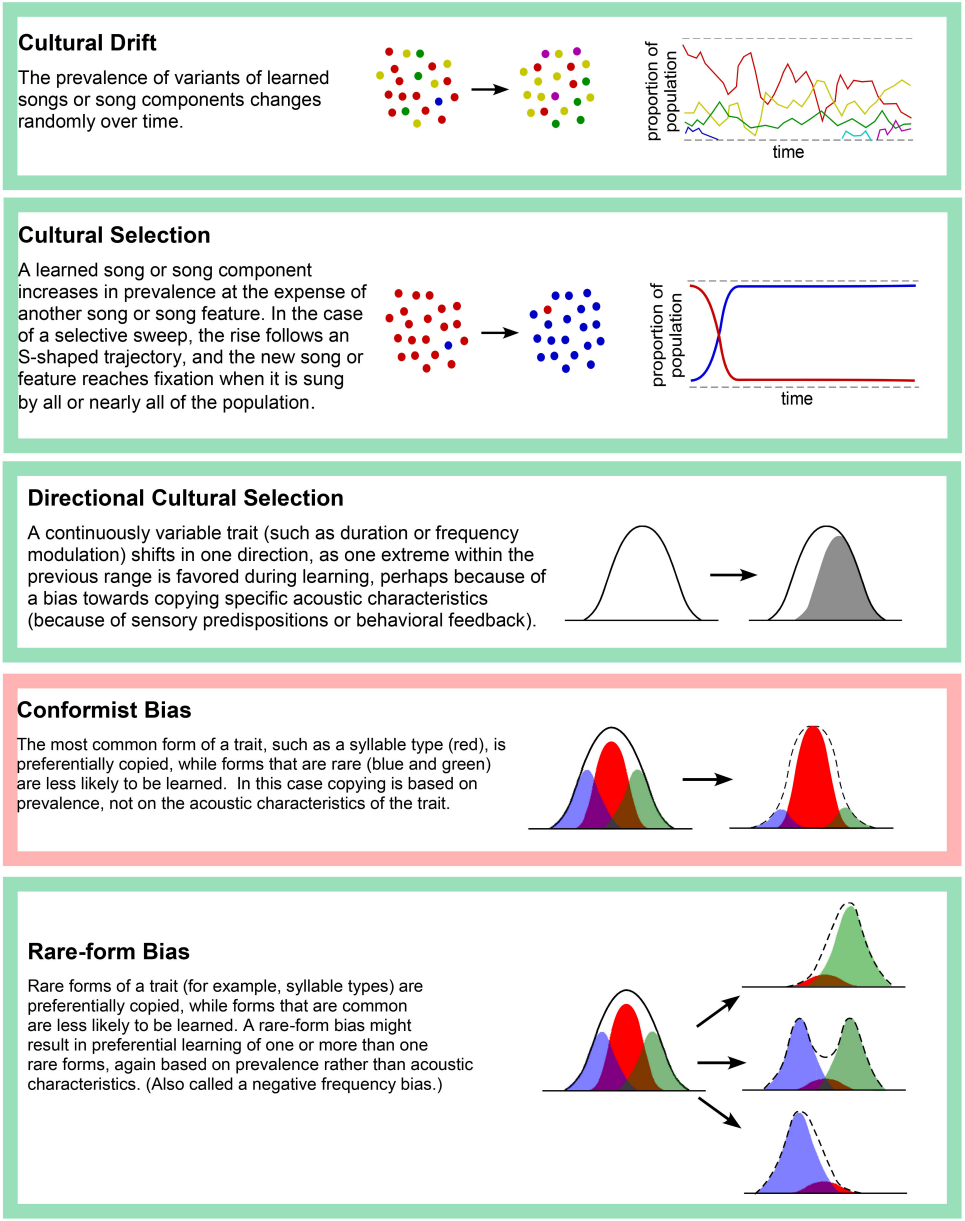

*Color coding of borders denotes whether the mechanism is likely to result in stability (red) or change (green). Selective mechanisms shown here can be driven by (1) sensory bias or motor constraints in the learner, (2) natural selection (such as change that increases the efficacy of signal transmission), (3) sexual selection, in the form of male-male interactions or female choice, as well as (4) demonstrator biases (copying songs of prominent males)*.

Copy errors, innovation, and improvisation insert novel variants into learned songs of wild populations at a relatively high rate: modeling studies have estimated that song “mutation rates” fall between 0.1 and 3.5% (e.g., Lachlan and Slater, 2003), and observational studies have yielded estimates of approximately of 5% (e.g., Lynch et al., 1989). Genetic predispositions may affect the incorporation of such variants into songs at the learning stage (Tchernichovski et al., 2017). However, only a subset of the wide range of “acceptable” species-typical song material is present within the songs of a single population. Mechanisms that promote stability act to restrict the number of a population's species-typical syllable or song variants, while mechanisms that promote variation result in expansion of or change in the population's species-typical repertoire.

### Stability

We can infer that maintaining long-term stability in a learned bird song or song segment is likely due to stabilizing selection, either because of ecological factors or because of learning biases. An ecological factor, vegetation that favors the transmission of specific acoustic characteristics (Morton, 1975), correlates with stable song features in the rufous-collared sparrow *(Zonotrichia capensis)*. Trill types are consistent within a habitat, and a male's trill type is related to the vegetation in his territory – and not to the altitude of the territory and so to physiology, or to the bird's genetic origin and hence to the songs it heard as a juvenile (Handford and Nottebohm, 1976; García et al., 2015). Such correlations allow us to infer that stable ecological constraints play an important role in driving stability of some song features, as young males in a population learn a habitat-appropriate song.

Learning biases may also result in song stability. Robert Lachlan and his colleagues used mathematical modeling to explore the maintenance of learned syllables in the songs of swamp sparrows *(Melospiza georgiana)*, which fall into clearly defined categories (Marler and Pickert, 1984). Both males and females respond more strongly to “typical” syllable forms, rather than to variants that have acoustic characteristics that differ from the population's average values (Lachlan et al., 2014). However, there are also “outlier” syllables within the songs of each population, suggesting that the consistency of syllable types within populations over time is likely to be due to cultural conformity. To test this hypothesis, Lachlan et al. (2018) modeled the distribution of syllable types within six different populations, testing the roles of mutation rate, demonstrator bias (copying high-ranking individuals), content bias (genetic predispositions), and conformist bias (copying the most common syllables) in maintaining stable syllable types. The model clearly supported a role for conformist bias in maintaining the most common syllable types. Lachlan et al. (2018) suggest that a conformist bias in swamp sparrow songs could be implemented by discarding songs that are not heard at or around the time of crystallization – “overproduction” during plastic song followed by “attrition” at the time of crystallization, as described by Nelson and Marler (1994). Since over-production followed by attrition also occurs during song learning in white-crowned sparrows (Nelson, 1992) and Savannah sparrows (Thomas et al., 2021), the stable segments of those species' songs might be subject to a similar conformist learning bias. However, the conformist bias model for maintaining stability of learned song segments for these two species is complicated by the presence of variable song segments within the same songs. Individual birds learning their songs would need to apply a conformist bias only to the song segments that are consistent and stable in the songs of adult singers. Young birds could assess the variability of each song segment while listening to adult singers during development (and again during the period immediately prior to crystallization) and then use a rule that applies a conformist learning bias only to song segments that have dominant forms within the population of adult singers. Such “conditional conformity” is more cognitively complex than a simple frequency-dependent learning bias: young birds would need to not only assess which is the most common form and copy that form, but would also need to determine which songs and song segments are *not* subject to a conformist bias by assessing the relative variability of those songs and segments. Tchernichovski et al. (2017) pointed out that different social networks may favor different patterns of variability. If different songs or segments are important in different social contexts, young birds would also need to weight the importance of variability in models' songs according to the responses of other conspecifics.

Songs or song segments that maintain the same form within a population – such as the trill of the white-crowned sparrow and the buzz of the Savannah sparrow – seem likely to be subject to conformist biases, whether simple or conditional. The appearance and transmission of novel variants of such song segments within a population may thus shed light on the mechanisms that promote stability. The acoustic features of the Kent Island buzz were stable between 1980 and 2014, as were those of a different population, in Williamstown, between 2008 and 2012 (Williams et al., 2019; Figures 2A–C). Within that overall pattern of stability, however, 8.9% of birds on Kent Island sang one of two variant forms that had lower mean frequencies (Figure 2A). These variant versions were first recorded in the Kent Island population in the late 1980s, and then fluctuated in prevalence during the 1990s (Figure 2D), suggesting that cultural drift was the mechanism driving the learning of this variant. In 2003, variant buzzes were sung by 25% of the population (with two birds singing both standard and variant forms). The most successful males on the study site included individuals singing buzz variants as well as those singing standard buzzes, so sexual selection is unlikely to have favored either buzz type. After 2003, the prevalence of variant buzzes declined rapidly, in a trajectory that is more suggestive of selection than drift. It is not clear why the variant buzzes disappeared, but there are two intriguing possibilities. One is that a common-form bias was triggered or strengthened when the rare form's prevalence drifted above a threshold (another version of a conditional frequency-based learning bias). A second possibility arises from the observation that the mean frequencies of buzzes sung in the Kent Island population during the 1990s fell within a continuous range. However, after 2000, buzz frequencies distinctly fell into either a high range or a low range (Figure 2B). Perhaps this split into high and low frequency ranges resulted in the formation of perceptual categories such as those described behaviorally in starlings *(Sturnus vulgaris*, MacDougall-Shackleton and Hulse, 1996) and which are discriminated by neurons in the auditory forebrain of swamp sparrows (Prather et al., 2009). Once the buzzes were perceived as two distinct categories, conformist learning would result in elimination of the rare form. Either the “prevalence threshold scenario” or the “categorical perception scenario” would add complexity to the standard formulation of frequency-biased learning.

### Change in Songs and Song Segments

#### Drift

As is the case for genetic evolution, change in a population's song may be due to processes that are not selective. Cultural drift, like genetic drift, results in random changes in the frequency of traits, sometimes culminating in elimination or fixation of those traits, and has a stronger influence when population sizes are small. Several studies have followed the cultural evolution of songs across chains of islands that have been colonized in succession, which makes it likely that cultural drift affected song traditions in the small founder populations.

Chaffinches arrived in the Chatham islands near New Zealand during the last 150 years, and populations on these islands sing a reduced set of syllables, all of which match syllables sung on the mainland (Baker and Jenkins, 1987). In this case, the population bottleneck that occurred at the time of colonization appears to have resulted in the loss of song material when some syllables were not learned by the next generations, reducing variation within the population, just as genetic drift reduces variation during a population bottleneck.

The rate of population growth that follows a population bottleneck and the resulting density may be related to whether variation is reduced, as in the Chatham island chaffinches, or, in a seeming paradox, increased. The chaffinch population in the Azores arose from mainland European populations within the last million years and, although the genetic structure of these island populations bears the signature of bottlenecks with each successive colonization event, populations are now large and dense (Marshall and Baker, 1999). Song variability is higher in the island populations than on the mainland (Lachlan et al., 2013). It is possible that, shortly after colonization, individuals hear fewer models and are thus more likely to improvise, innovate, or learn inaccurately. Lachlan et al. suggested an explanation: when a population is small and sparse, individuals are less likely to encounter conspecifics, and species-specific learning biases may be relaxed – allowing any novel song forms that arise because of a higher “mutation rate” to be recognized as appropriate signals and then persist via social learning.

Increased variability as a consequence of cultural drift also occurs in the chickadee *(Poecile atricapillus)* “fee-bee” song. This “standard song” is consistent across North America – except in a few island populations at the edges of the continent and in areas where habitat is scattered and sparsely populated (Kroodsma D. E. et al., 1999). Individuals in small populations occupying habitat islands near Fort Collins, Colorado sing variants as well as the standard song (Gammon and Baker, 2004). These variants appear to have arisen from juveniles' improvisations; recordings of young birds singing prior to crystallization include variation beyond that sung by typical adults but similar to the songs in the habitat islands (Gammon et al., 2005). These results fit the conception that small, sparse populations favor the relaxation of learning constraints, allowing a wider range of signals to be recognized as conspecific and so avoiding the exclusion of individuals with song variants from the breeding population because of their unusual songs.

House finches provide another example of cultural drift of songs in large populations. After being introduced to the New York City area in the 1940s (Elliot and Arbib, 1953), house finches spread more than halfway across North America by the late 1980s. Songs from the leading edge of this invasion show trends similar to those seen in the chaffinches of the Chatham Islands, with new populations singing a subset of syllables found in the source populations (Tracy et al., 2009). Ju et al. (2019) compared songs recorded near New York City in 1975, when the population was already large, to those recorded in the same areas in 2012. Both song types and syllable types largely turned over during that 40-year span. Syllables that were common in 1975 songs were more likely to persist in 2012 songs (although they were not necessarily still common in 2012). Measurements of acoustic parameters did not predict which of the 1975 syllables persisted until 2012, suggesting that not selection but drift – in the form of random loss of rare syllables and introduction and transmission of novel syllables – explains this pattern of house finch syllable loss and gain.

**Figure 3:**
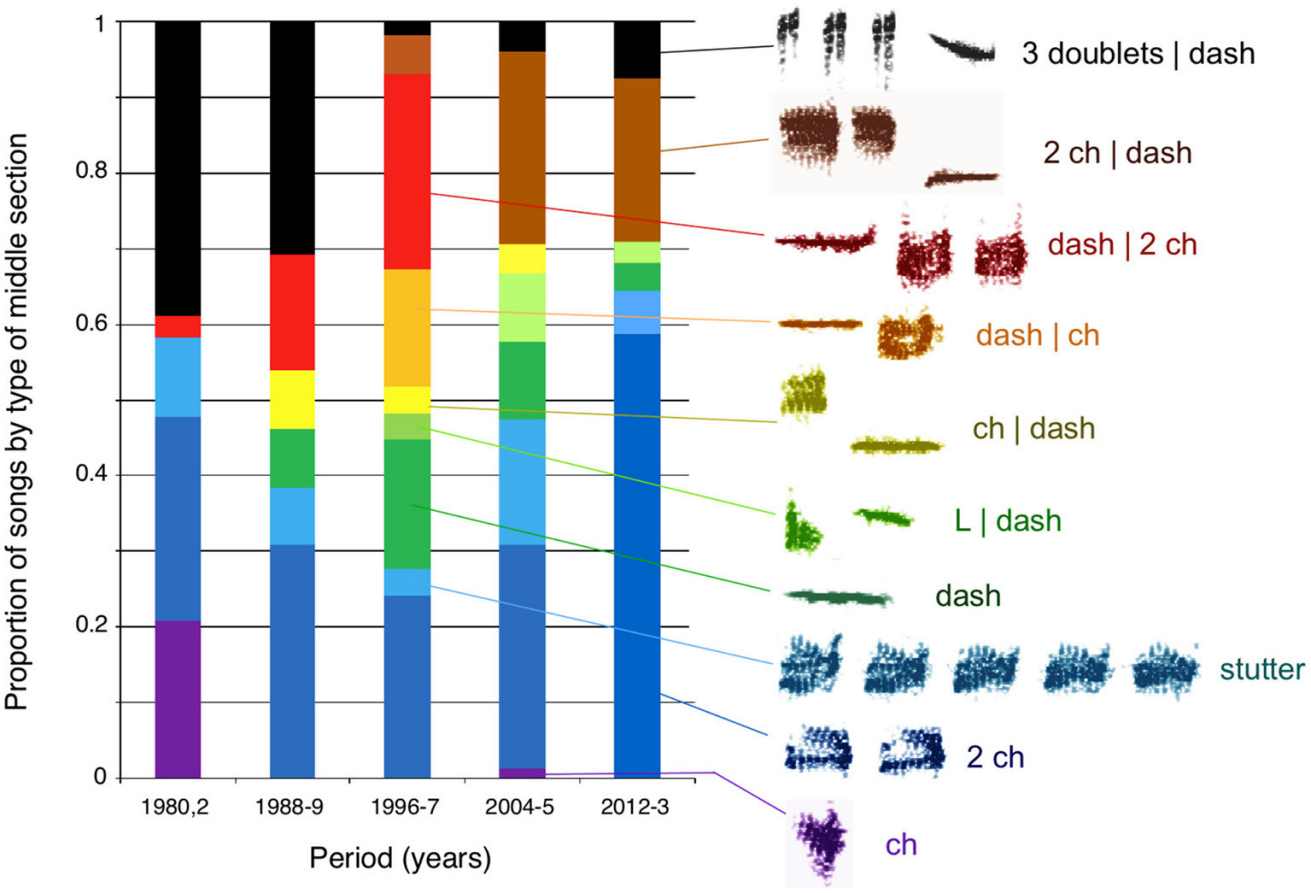
Changes in the prevalence of types of middle sections of Savannah sparrow song. Middle section types were defined based on the type, number, and order of syllables. The two main classes of notes in middle sections are “ch” notes (noisy, with multiple frequencies), and “dash” notes (tonal). Three types of middle section were composed solely of ch notes, differing in the number of notes, either 1 (violet), 2, (dark blue), or >2 (a “stutter,” light blue). Dash notes (dark green) made up the middle section or were combined with one or more ch notes, with the dash note falling in either the first or last position (brown, red, orange, and yellow). An “L” note (modified low-frequency ch note) followed by a dash (light green) formed another category. Noisy doublets followed by a dash (black) made up the final category plotted here. For the purposes of clarity of illustration, some categories that are split elsewhere are combined here (After Williams et al., 2013).

Cultural drift is also responsible for the rapid turnover of syllables within the unaccented songs of chestnut-sided warblers. The distribution of syllable types recorded during any one time period follows a power law, as would be expected if syllable prevalence is determined by random processes (Byers B. E. et al., 2010). The apparent stochasticity of change in the prevalence of note complexes of white-crowned sparrow songs and middle section types of Savannah sparrow songs (Figure 3) raises the possibility that notes and clusters of notes in these song segments also turn over because of cultural drift. Comparisons of power law distributions to the patterns of note type prevalence in the variable note complex segment of white-crowned sparrow songs and middle segment of Savannah sparrow songs (Figure 3) could be used to test the hypothesis that the variability we see in those song segments is due to stochastic processes and so represents cultural drift.

The term “drift” emphasizes that change is due to random processes, but cultural drift may in some sense be an active process in repertoires that include both stable and variable songs or song segments. Partitioning of drift and selective processes could be based on innate mechanisms that define the degree of variability in specific song types or song segments. However, songs or song segments might be “designated” as fixed or variable because young birds apply specific learning rules based on the patterns of variability they observe in the population. To learn which song segments are variable and which are fixed young birds would need to hear the songs of a relatively large number of adults, either within a dense breeding population, or during out-of-season singing when birds are not territorial and may gather in groups. Whether genetic influences or experiential learning determine where to apply learning biases and so define which song types or segments are subject to cultural drift is a question for future research.

#### Learning Biases

As they apply to bird song, learning biases that shape the outcome of song acquisition might be based on (1) genetic predispositions, (2) motor or sensory constraints, (3) model (or demonstrator) status, or (4) prevalence (as a bias toward copying either a common or rare song feature). Peter Marler's foundational work on bird song learning demonstrated that innate biases cause young birds to (a) generate species-specific song features in the absence of a model (Marler and Sherman, 1983, 1985), and (b) when a choice of models is available, preferentially copy song features with acoustic characteristics that match a genetic template (Marler and Peters, 1988). Part of the basis for such preferential learning of species-specific song may lie in biomechanical or motor constraints that make certain acoustic features easier or more difficult for the vocal apparatus of a given species to produce (reviewed in Podos et al., 2009), or in sensory predispositions that make certain acoustic features more likely to be favored (see Sakata and Yazaki-Sugiyama, 2020). However, birds of many species can learn atypical songs if the only live song model they hear is not a conspecific; for example, zebra finches, which normally sing and prefer songs rich in harmonics, will produce an accurate copy of a strawberry finch's *(Amandava amandava)* tonal song if they are fostered into a strawberry finch nest (Immelmann, 1969). This review concentrates on the role of social factors that affect song learning outcomes rather than on genetically-based factors.

##### Demonstrator Biases

Young males may copy the songs of older males with particular characteristics rather than basing their choice of a model on the acoustic characteristics of the song. Males that have survived longer, are dominant, have larger territories, attract more females, have more offspring, or are good foragers and provide more provisioning to their offspring might have special salience for the young males in the next generation, potentially making those salient males' songs more likely to be copied. The acoustic characteristics of the songs of prominent models may also denote physical or developmental advantages, as they are influenced by the physical characteristics of his vocal tract (Ryan and Brenowitz, 1985; Derryberry et al., 2018; Garciá and Tubaro, 2018). Because many species follow an overproduction/attrition learning trajectory, preferential copying could take the form of (a) memorizing the salient adult's song during a young male's first summer or (b) retaining a song that matches a prominent male that is present during the later “attrition” phase of song learning. Young birds that have multiple opportunities to assess adult singers during song learning are more likely to employ these strategies.

Indigo buntings *(Passerina cyanea)* provide evidence for this type of model bias: older males, which have brighter plumage, are copied more often than young males (Payne et al., 1988). Perhaps the most compelling evidence for a model-based song learning bias comes from studies of cowbirds *(Molothrus ater)*. Cowbirds are brood parasites and often form groups of males courting a single female, allowing young males to observe and assess songs as well as behavioral outcomes for multiple adult males simultaneously. As young males do not crystallize their songs until their 2nd year, they have ample opportunity to observe that females prefer to mate with older males singing the local song type, and young males shape their songs accordingly to match those of successful older males (O'Loghlen and Rothstein, 2003). In territorial species, young males may be able to observe the dominance, mating success, and reproductive success of neighboring adult males either directly or by means of cues present in singing behavior. For example, males may sing frequently during periods when others are silent if they do not have mates (Liu, 2004); songs that overlap in time may signal aggression and dominance (Mennill et al., 2002); females' displays and calls may signal receptiveness (MacDougall-Shackleton et al., 2001); and males may sing a few times from the nest or the fledglings' location as they feed young (Wheelwright and Rising, 2008). Although we do not yet know how or whether model characteristics affect song learning in Savannah sparrows, these behaviors may potentially provide important information about adult male sparrows' status and success and allow young birds to preferentially learn songs associated with high-ranking, successful males.

##### Frequency-Based Learning Biases

Preferential learning of common or rare songs or song segments can also result in selection that drives cultural evolution. Conformity biases promote stability (see above), while rare-form biases result in preferential transmission of novel or unusual songs. However, cultural drift (see above) or a song development program that favors innovation may also result in variation and rapid change of song types. Nomadic North American sedge wrens *(Cistothorus stellaris)* take the production of novelty during song learning to an extreme: they do not copy songs. Instead, each young bird develops its song *de novo* within species-specific parameters (Kroodsma D. et al., 1999). Although improvisation or innovation as a song learning strategy clearly favors variation and change in this species, it is not a form of frequency-biased learning, as songs are not copied. In contrast, a rare-form learning bias occurs when young males preferentially copy songs or syllables that are currently sung by relatively few adults in their population. In the laboratory, Tchernichovski and Nottebohm (1998) found that if several young zebra finches were exposed to the same song model, the first males to crystallize their songs produced the most accurate copies of the model, increasing its prevalence in the experimental population. Late crystallizers were more likely to alter the model's song or add novel syllables. One possibility is that late crystallizers are less likely to learn an increasingly common song (Tchernichovski et al., 1999); alternatively, early crystallizers might have a developmental trajectory that favors more accurate copying for neural or physiological reasons. Another laboratory study of zebra finch song learning (Fehér et al., 2009) noted that syllables that are repeated several times in the model's song tend to be repeated fewer times in the copied song, a variation on the theme of frequency-dependent learning. Perhaps what we conceive of as a rare-form preference would be more properly termed “common-form avoidance.”

Although rare form preference in song learning has been the subject of a number of laboratory and theoretical studies, compelling examples from wild populations are relatively rare. Grant and Grant (1996) pointed out that female *Geospiza fortis* (Darwin's medium ground finch) tend to avoid mating with males whose songs are similar to those of the females' fathers; this pattern would favor copying of rare songs to provide males with a wider range of potential mates. Goodale and Podos (2010) proposed that such a rare form preference explained the novel songs they observed in a population of *G. fortis* recorded in 1961 and then in 1999. In such studies, distinguishing between the turnover that results from cultural drift and the changes that arise from a rare-form learning bias can be difficult. The result of a rare-form learning bias should be more rapid turnover of song or syllable types than would occur if cultural drift alone were operating. One possible method for distinguishing between the turnover of songs or syllables that results from cultural drift and the turnover that results from a rare-form bias might be is the observed probability that novel (and thus by definition, rare) songs or syllables increase in prevalence within a population's songs. If random processes are at work, novel syllables or songs should be equally likely to disappear or to increase in prevalence over the next few generations. On the other hand, if a frequency-based bias for learning rare syllables or songs is driving change, novel forms should increase in probability more often than would be predicted by chance. Long-term recordings from a population with variable songs or song segments, such as the note complex of white-crowned sparrow song and the middle section of Savannah sparrow song, may provide an opportunity to document possible rare-form biases.

A frequency-based learning bias need not be absolute. An interesting combination of variation and stability occurs in the yellow-rumped cacique *(Cacicus cela vitellinus)*, which have songs that change gradually over each singing season – but the changes are synchronous within a population of singers (Trainer, 1989). Here a conformity bias appears to keep the evolving song matched within the population, but allows for incorporation of novel (rare) song elements if they are quickly adopted by all or most of the members in a group. In European nightingales *(Luschinia megarhynchos)*, both rare- and common-form frequency-based learning biases may operate at the same time. The nightingales have large song repertoires that include both widely shared songs, perhaps due to conformist copying, and rare songs, perhaps due to a rare-form learning bias (Sprau and Mundry, 2010). The ability to produce both rare and common songs would allow an individual to match other males' songs and also to make it difficult for other males to match them. Research on western song sparrows *(Melospiza melodia)* points out the importance of song matching; during the late stages of song learning song sparrows retain songs that are sung by their neighbors and so can be used to match those neighbors during countersinging bouts, yielding a system that differentiates signals of intention based on whether a male (aggressively) matches the song his neighbor is singing or avoids matching that song (Beecher, 2017). Learning of both common and rare song forms would be favored in species that use song matching to signal aggression. However, a dual frequency-based learning bias adds cognitive complexity: it would require young birds to track the prevalence of all song forms, and then apply two thresholds (one defining rare songs, and another defining common songs) to determine which songs to copy.

#### Content-Based Selection

In contrast to learning biases, which favor the copying of variants based on the attributes of the singers (dominance, success) or of the prevalence of song variants within a population (frequency-based learning biases), content-based selection favors the learning of songs or syllables with specific acoustic characteristics (Podos, 2001). Learners may have a sensory predisposition that favors learning one song form or one type of acoustic feature. On the purely physiological/morphological level, larger individuals may be better able to produce lower frequencies, and males with smaller beaks may be able to more rapidly modulate frequencies (Westneat et al., 1993; Derryberry et al., 2018; Garciá and Tubaro, 2018). Individual differences in motor constraints arising from developmental variation in vocal organs or neural circuits controlling vocalization (as described by Nowicki et al., 2002a) also shape song learning.

Some song features may be preferentially copied not because of constraints or predispositions specific to the individual copier but because of selective advantages that those features provide to the singer. Changes in a population's song that are driven by natural selection should result in adaptation to environmental conditions, and changes that are driven by sexual selection should result in greater reproductive success due to an advantage in male-male interactions or in female choice. Evidence for selection, whether natural or sexual, may come in the form of a selective sweep, a rapid, continuous replacement of one form by another – in contrast to the slower, more erratic trajectory of changes due to cultural drift.

##### Natural Selection and Adaptation

Selection on learned songs should result in adaptation: over time, selection favors a song that confers an advantage to individuals who sing that song, because the signal is more effective. Changes in response to environmental noise provide the clearest examples of selection on and adaptation of a learned song. Anthropogenic noise, particularly noise in urban environments, results in auditory masking of song components, especially those including lower frequencies. An upward shift in the frequencies of songs of birds in urban areas has been documented in several species (see Slabbekoorn, 2013). One example is the dark-eyed junco *(Junco hyemalis)* population in the San Diego, CA area, which has higher-frequency songs than does a population in a nearby forest. This difference is due to both an upward shift in the song types that birds in the two habitats have in common, and to urban birds replacing lower-frequency songs with different higher-frequency songs (Cardoso and Atwell, 2010). Interestingly, such an upward shift in the frequencies sung by urban birds may reduce the ability of the singers to differentiate their songs in terms of some acoustic parameters, such as frequency bandwidth, that females use as the basis of mate choice. Apparently to compensate, males increase vocal performance in other acoustic characteristics, such as frequency modulation (Moseley et al., 2019). Derryberry et al. (2020) and her colleagues confirmed that anthropogenic noise drives upward frequency shifts in urban bird by measuring noise levels and song frequencies of white-crowned sparrows in San Francisco, CA during the coronavirus shutdown of 2020. Anthropogenic noise decreased, reverting to levels present in the 1950s, and birds responded by singing songs at lower amplitudes and with greater bandwidth – a very rapid adjustment to changes in selective pressures on song characteristics. Evidence for adaptation in the acoustic properties of songs after change in habitat characteristics is not restricted to urban environments. Silvereyes *(Zosterops lateralis)*, a species found in Australia, have colonized islands in the Tasman Sea between Australia and New Zealand; sharing of syllables between populations decreased with each colonization step, and this cultural drift was well-predicted by measurements of genetic drift in recently diverged populations (Potvin and Clegg, 2015). However, the acoustic characteristics of songs sung by older populations were best predicted by habitat type and ambient noise; as time passed, selection by the acoustic environment resulted in adaptive shifts in song characteristics. The silvereye example reinforces the importance of being aware that multiple mechanisms may act simultaneously or sequentially to shape the cultural evolution of bird songs.

##### Sexual Selection

As is the case for genetic evolution, environmental characteristics are not the only selective mechanism influencing the cultural evolution of learned songs. The outcome of bird song learning may be influenced, mediated, or driven by both the intersexual and intrasexual components of sexual selection (in species that are the focus of this review, where males are the primary or only singers, sexual selection takes the form of male-male interactions and mate choice by females). Song learning can be shaped by both modes of sexual selection. In some cases, the two modes are linked, but in others, they may act in opposition and so stabilize song features at an equilibrium or drive different song features in different directions. Although many papers cover the potential role of sexual selection in the evolution of song learning (e.g., Nowicki and Searcy, 2014), relatively few directly address the question of how sexual selection shapes cultural evolution. Those that do generally use one of two methods: (a) correlations between song features and reproductive success, and (b) playback studies that test birds' responses to specific song features. Both of these methods are useful. However, they provide a measure at a specific time point, and both the landscape of song that sexual selection acts upon and the preferences that result in sexual selection evolve.

Many aspects of song performance appear to be important in male-male interactions (Byers J. et al., 2010), and responses to playback stimuli have helped to define the roles of particular song segments or acoustic characteristics in conflicts between males. Specific acoustic features such as faster trill rates and greater frequency bandwidth are more effective at deterring approach by other male banded wrens (*Thryothorus pleurostictus*; Illes et al., 2006; de Kort et al., 2009). Alternatively, matching the songs of neighbors allows western song sparrows to signal varying degrees of aggression (Akçay et al., 2013). Such song sharing appears to benefit both an older male that serves as a song model and a younger male neighbor that copies the song (Beecher et al., 2020). Local versions of the Savannah sparrow buzz song segment, which is stable and population-specific, elicit the strongest aggressive responses from males (Williams et al., 2019; Figure 2E). Similarly, white-crowned sparrow males also respond more aggressively to local trills – again, a stable and population-specific portion of the song (Nelson and Soha, 2004b). In contrast, the note complexes of white-crowned sparrow song vary across individuals, and males respond more aggressively to strangers' note complexes than to those of neighbors (Nelson and Poesel, 2007), suggesting that the variable middle segment of Savannah sparrow songs might also be important for individual recognition. Sexual selection driven by male-male interactions may thus favor (a) acoustic characteristics that play a role in deterring intrusion by other males, (b) the learning of shared songs that can be used to negotiate aggressive interactions, (c) stability of song elements that denote population identity, and (d) variable song elements that allow for individual identification. Male-male interactions could thus favor a variety of mechanisms that promote stability, random change, or directional change within a population's song.

The second component of sexual selection, female choice, is also likely to play an important role in the social learning of song and consequently in the cultural evolution of song. In the laboratory, female canaries are especially responsive to song phrases that include short silent intervals and sharp frequency drops (Kreutzer and Vallet, 1990), although songs of male canaries include other phrase types as well. Also in laboratory studies, female responses to young males' plastic songs guides vocal development in cowbirds (West and King, 1988) and zebra finches (Carouso-Peck and Goldstein, 2019). Depending upon the species and the behavior measured, wild females prefer the local song (e.g., cowbirds, O'Loghlen et al., 2011) or do not differentiate between local and foreign songs (e.g., white-crowned sparrows, Nelson and Soha, 2004a). In a number of studies, both in the laboratory and in the field, males singing in a courtship context use specific songs or song structures that differ from those used when singing alone or to other males (e.g., zebra finches, Sossinka and Böhner, 1980 and Sakata et al., 2008; chestnut-sided warblers, Kroodsma et al., 1989; house finches, Ciaburri and Williams, 2019), suggesting that such songs or song segments are subject to female choice and so evolve based on females' preferences.

Among the acoustic characteristics that correlate to measures of success are the trills of male Savannah sparrows in Ontario: males that sang double rather than single trills arrived on territories earlier in the breeding season and fathered more fledglings (Sung and Handford, 2020). If the prevalence of songs with double trill endings increases in this population, sexual selection would be implicated. In the Kent Island population of the same species, males that sang click trains (see Figure 1) as part of their introductory segment in 2003–4 produced more fledglings, and the prevalence of click trains subsequently increased (Williams et al., 2013). For this case the next step in the logic of demonstrating sexual selection has been satisfied, but we do not know whether the advantage to click trains lies in sexual selection based on male-male interactions or on female choice; only playback experiments performed in the early 2000s could answer that question. Female Savannah sparrows return to breeding areas later than males in the spring (Wheelwright and Rising, 2008), but most 1st-year males will have crystallized their songs before females arrive (older males do not change their songs) so female responses are unlikely to affect the attrition phase of song learning in this population. However, a young male can observe females' responses to adult males' songs during the previous summer and such observations may guide the early stages of song learning. In species that do not migrate, females may affect all phases of song learning.

The differences in male and female responses to acoustic and structural song features implies that the effects of sexual selection on song learning vary and are likely to be specific to particular songs or song segments that pertain to the social context. Where the same song is important both for female mate choice and in contexts that involve male-male interactions (such as territorial advertisement), it can be hard to define the effects of sexual selection on song transmission. Developing experimental designs that tease apart the valence and strength of male and female responses to specific songs, song segments, and syllables will be important for understanding the effects of sexual selection on song learning and the implications for cultural evolution.

##### What Is Under Selection: The Singer or Song?

Studies of cultural evolution may find it difficult to disentangle the acoustic characteristics of the song from the physical and social characteristics of the singer. For example, consider the chickadee's gargle call, a complex learned vocalization that is distinct from the fee-bee song. The gargle is used during aggressive interactions, and changes over time (Baker and Gammon, 2008). Cultural drift would seem to be an important factor in the turnover of gargle calls within a population, as calls that are restricted to the repertoire of only one male are the most likely to be lost in the next generation. Acoustic characteristics also play a role, with shorter, lower-pitched calls more likely to be lost. Demonstrator bias may also be a factor, as gargle calls are often used by birds interacting in winter flocks, when body condition and dominance status are readily apparent; calls that persist from year to year are more likely to be in the repertoires of older birds in good condition. Are gargle calls that persist in the population copied because their acoustic parameters are under selection for improved function? Because they are more common and less subject to loss via cultural drift? Because males of higher social status are preferentially copied? Because females prefer longer, higher-pitched calls? As long-range communication signals, the acoustic characteristics of a song, as well as its prevalence within the population, have a perceptual existence separate from the singer. However, at short range the song is perceptually linked to the singer.

Disentangling the many influences on the social learning of songbirds' vocalizations will require carefully designed experiments and analyses. Other factors beyond those considered in the analysis of chickadee gargle calls further complicate the picture. Sensory biases, such as those described in the auditory forebrain of swamp sparrows (Prather et al., 2012) may favor song characteristics that do not denote male quality and do not confer a selective advantage to the singer, and so influence cultural evolution based solely on the acoustic characteristics of the song (as is also the case for environmental effects on signal transmission; see above). Environmental factors, such as the feeding regimes experienced by young birds, may affect song development and so make the quality of song a potentially unreliable predictor of male quality (Nowicki et al., 2002a), yet females may still prefer more accurately learned songs (Nowicki et al., 2002b). Female choice can also be based on male characteristics other than song or in addition to song. For example, a zebra finch male's beak coloration appears to be more important than his song from a female's perspective (Simons and Verhulst, 2011), and compatibility of personality traits is important for both female choice (Schuett et al., 2011a) and the ensuing reproductive success of a pair (Schuett et al., 2011b). A zebra finch male might then transmit his song to his own and to other offspring through demonstrator bias, and if young females associate that song with a successful male, the song's success would be secondary to the singer's beak coloration and behavioral traits. Only a more complete understanding of the relationship between the singer's and the song's characteristics, and how those characteristics affect song transmission, can fully resolve the question of whether a signal that functions in mate attraction is under selection – or whether other aspects of the singer result in his success, and only secondarily in the success of the song he sings.

##### Selective Sweeps and Directional Selection

One signature of a trait that is under positive or directional selection is a selective sweep, a rapid and continuous increase in the frequency of a trait within the population from a very low level immediately after introduction of the trait to fixation (present in all or nearly all of the population). In studies of human cultural evolution, selective sweeps are often described as “S-shaped curves” or, in the case of successive selective sweeps, “battleship curves,” and these characteristic trajectories of change in prevalence are also seen in the replacement of some learned song features in wild populations of songbirds. In North America, the white-throated sparrow *(Zonotrichia albicollis)* has a distinctive whistled song often described with the mnemonic “Old Sam Peabody Peabody Peabody”: two or three initial notes with variable frequency followed by a series of triplet notes at the same frequency. However, a variant with doublets instead of triplets (perhaps calling for a revised mnemonic of “Old Sam Peady Peady Peady”) arose in the northern Rocky Mountains sometime between 1960 and 2000 (Zimmerman et al., 2016). The doublet form has spread rapidly eastward since 2000, is now sung over most of North America, and appears to be continuing its eastward spread into areas where triplets are still sung (Otter et al., 2020). The question of why the doublet is preferred to the triplet form during song learning is currently open, and could be addressed by studies that play both forms to males and females of populations with different proportions of the triplet form. The timing of such a study is crucial. Results may be very difficult to interpret if the population has only experienced one song variant in its natural environment – but selective sweeps may not be well-documented until after they are complete. The ongoing replacement of triplet songs by doublet songs in white-throated sparrows makes this system particularly interesting.

**Figure 4:**
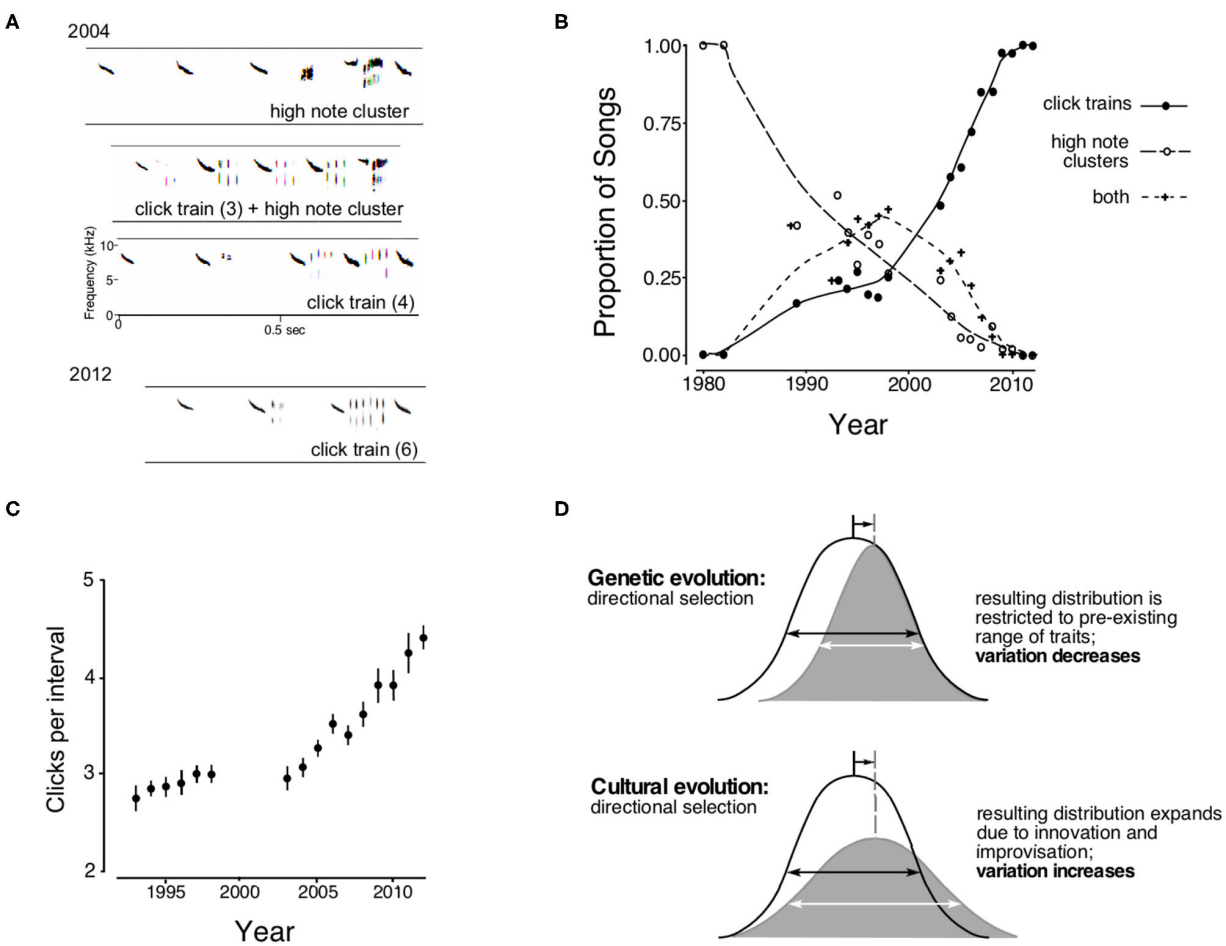
Cultural selection: the interstitial notes of Savannah sparrow song. **(A)** Examples of introductory segments. In 2004, birds sang interstitial note sequences consisting of either high note clusters (top), click trains (bottom), or both types in succession (middle). In 2012 high note clusters were no longer present, and the number of clicks within an interval between introductory notes had increased. **(B)** The trajectory of high note cluster replacement by click trains, first sung in tandem and then as click trains alone. The curves are fitted splines. **(C)** Average number of clicks per interval between introductory notes for songs that included click trains (error bars represent the SEM). **(D)** Schematic representation of the effect of directional selection in genetic and cultural transmission. The population mean increases by the same amount for each type of selection. Horizontal double-headed arrows represent the corresponding original (black) and new (white) standard deviations (After Williams et al., 2013).

The white-throated sparrow doublet song changes were observed as a geographical phenomenon by recording birds at different locations, but selective sweeps can also be observed in long-term studies of a single population. The introductory portion of the Savannah sparrow song is dominated by an accelerating series of loud, high-pitched, descending “introductory notes” that are consistent across populations (Wheelwright and Rising, 2008; Wheelwright et al., 2008). In the intervals between those notes, particularly in the final intervals before the transition to the next song segment, birds sing low-intensity “interstitial notes” that are usually consistent within a population (Williams et al., 2013). In recordings from the early 1980s, the dominant form of these interstitial notes in the Kent Island Savannah sparrow population was a three-part sequence consisting of (a) a brief series of similar, short notes; (b) a high-frequency (>9 kHz) tonal note; and (c) another brief series of short notes or a short trill (Figure 4A). As all the elements of this sequence have dominant frequencies above 7 kHz, this complex of interstitial notes is called the “high note cluster.” Another form of interstitial notes, the “click train,” consists of a sequence of 2–4 very short (3 ms) clicks and was first recorded in the late 1980s. In the 1990s, many birds sang both click trains and high note clusters in successive intervals between introductory notes. The prevalence of high note clusters steadily decreased and by 2010 click trains had altogether replaced high note clusters (Figure 4B). The higher reproductive success of males singing click trains in 2003–4 may have driven the sweep that resulted in the shift to click trains. However, we do not know the basis of this selective advantage – whether it had to do, for example, with signal efficacy, or was correlated to male dominance, or was favored by female sensory predispositions. Unfortunately, the fixation of click trains as an interstitial note trait makes it impossible to do meaningful playback experiments that compare responses of click trains to high note clusters (which would be a novel trait for the current population). Nonetheless, the rapid and continuous S-shaped trajectory of high note cluster replacement by click trains suggests that a selective sweep took place.

Click trains, although consisting of only one note type, vary in the number of clicks that are uttered in each interval between introductory notes. Between 1993 and 1995, the mean number of clicks per interval was 2.8; the number of clicks later increased, averaging 4.2 between 2010 and 2012 (Williams et al., 2013; Figure 4C). The trajectory of this increase suggests that directional selection is responsible. Further, the rapid expansion beyond the initial range of clicks points out an important difference between genetic and cultural evolution. In genetic evolution, selection for larger values of a trait decreases variation because, after selection, only individuals with larger values within the initial range are present. In contrast, individuals that acquire a trait through social learning may improvise on what they have learned and so go beyond the values present in the previous generation. The shift in the mean value of the cultural trait is thus due in part to an increase in the maximal values for the trait, and the trait's range expands rather than shrinking (Figure 4D). In the case of click trains, young birds that copied singers of 2–4 clicks could (and did) add additional clicks during song development, and the coefficient of variation for the number of clicks in the population increased at the same time as the mean increased. An increase of variation that coincides with a systematic shift in the mean value of a trait may prove to be a signature of directional cultural selection.

## Experimental Studies of Cultural Evolution in the Wild

**Figure 5:**
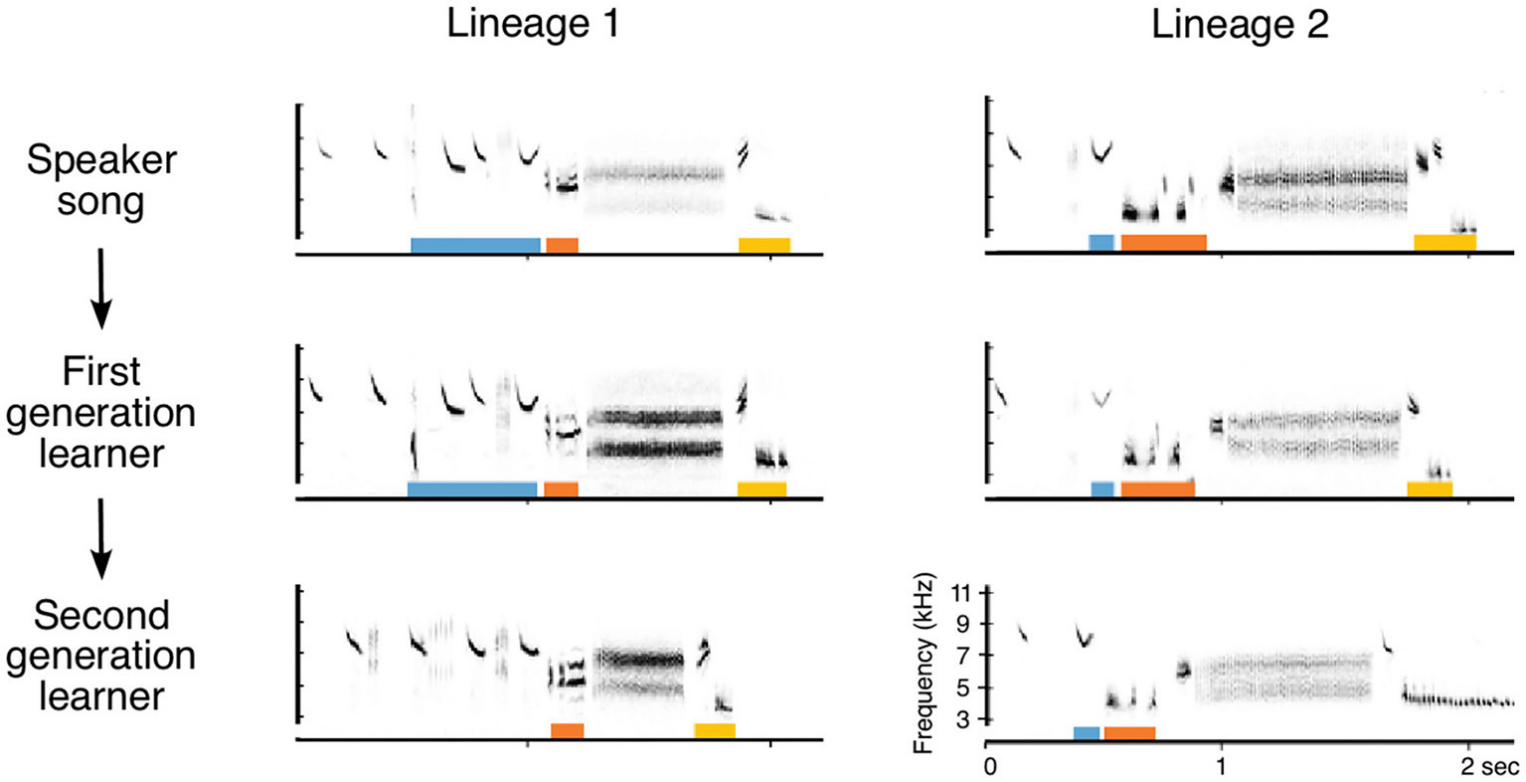
Transmission of seeded songs in a wild population of Savannah sparrows. Songs including characteristics not recorded in the previous 35 years on Kent Island (colored bars below notes on sonograms) were presented through an array of speakers across several hectares of Savannah sparrow habitat in the summer and subsequent early spring. Different songs were used each learning cycle, so birds that were older than the first-generation learners could not have learned the songs from speakers. The patterns of transmission of the novel seeded songs can be followed (After Mennill et al., 2018).

In addition to the methods traditionally used to study cultural evolution in populations of wild song birds (recordings from different geographical areas, recordings of a single population over time, sophisticated methods of acoustic analysis, mathematical modeling, and playback studies), experimental “seeding” promises to provide valuable insights. In seeding studies, a naïve individual is taught a novel behavioral variant, and the transmission of the seeded behavioral variant is tracked. This method has figured prominently in field studies of the cultural transmission of foraging techniques in songbirds (Aplin et al., 2015) and primates (Van De Waal et al., 2013), as well as in studies of song transmission in the laboratory (Williams et al., 1993; Fehér et al., 2009; Diez and MacDougall-Shackleton, 2020). Although immigration by individuals singing different songs can provide a natural seeding experiment, variables such as the timing and number of immigrants and the acoustic characteristics of the song that is introduced are not controlled. In the seeding experiment that was initiated in the wild Kent Island Savannah sparrow population in 2014, a distinct set of foreign songs was presented through an array of loudspeakers on the study site during each song learning cycle (Mennill et al., 2018). Some of the seeded songs have been transmitted across generations, as young hatched in following years learned them from live birds that had copied the songs played during earlier learning cycles (Figure 5). Interestingly, one feature of song performance that might provide salience for a model, simultaneous singing vs. solo singing, did not affect learning, suggesting that simple acoustic signatures of social interactions may not affect model salience (Mennill et al., 2019). The study continues, and promises to provide leverage for addressing questions about how the acoustic characteristics of the song and the characteristics of the singer affect transmission of that song, as well as insights into the possible differences in transmission mechanisms for stable and variable song segments.

## Conclusions and Areas for Further Study

The study of social learning of bird song has a long history of cross-pollination of field and laboratory studies. Peter Marler's observations of song learning and song dialects first gave rise to the idea that local song traditions arise from cultural evolution. Comparisons of songs of recently introduced populations (especially on islands) to source populations further suggested that song traditions change in ways that are analogous to genetic evolution. Extensive recordings from single populations across decades have allowed direct observation of changes that describe how song cultures evolve and provide evidence for a diversity of outcomes and mechanisms.

Different songs and different segments of a single song may evolve independently, with some remaining stable over decades and others changing rapidly. Conformist learning biases explain stability in some songs, such as the syllable types of the swamp sparrow, but are not entirely consistent with others, such as the buzz segment of Savannah sparrow song. Nightingales' song repertoires may be shaped by both common- and rare-form frequency-based learning biases.

Recently established populations may either have more diverse or more depauperate songs than ancestral populations. Population bottlenecks reduce diversity in newly colonized areas via cultural drift. On the other hand, in small populations, adult songs may retain some of the variety that is normally eliminated from juvenile songs through attrition-based conformity. Increased variation coupled with the relaxation of innately specified learning biases that is necessary to maintain a larger effective population size may also perpetuate the increase in the variety of syllables and songs.

Cultural selection may take several forms. Environmental (natural) selection, in the form of habitat that filters or ambient noise that masks acoustic characteristics, results in adaptive shifts in song characteristics. Directional selection results in a shift in the mean value of a song trait, and, since learning allows for change beyond a previous range of a trait, a signature of directional selection in cultural evolution may be an increase in the variation of the trait under selection. Learned song is important for male-male interactions and for mate attraction, so cultural sexual selection, in the form of preferential learning of song features that confer advantages in male-male interactions or in female mate choice is likely to play an important role. All of these learning biases and modes of selection may operate simultaneously to affect a song or song segment (perhaps leading to tradeoffs in acoustic characteristics), or may operate independently on different song features (particularly in the case of the two modes of sexual selection).

The basis for the success of a learned song trait is not always clear. Some males may be particularly attractive to females because of traits unrelated to their vocalizations, and young males that copy a dominant or successful male's song increase the song's cultural “success” even though the song itself has not being chosen. The importance of the relative roles of the singer and the song in the process of song transmission is a knotty problem and one that may have different answers for different species and different songs or song segments.

Dramatic changes in song cultures, resulting in selective sweeps where one song trait is replaced by another over a relatively short period, have been observed in wild populations. Ongoing selection events provide an important but time-constrained window for learning more about the factors that drive the adoption of a new trait at the expense of a previous form.

Learning biases are usually framed as simple gradients, favoring the copying of a more common song, or of the song of a more successful model. We need to consider the possibility that biases may be more cognitively complex. Young birds may first evaluate the relative variability of different parts of a song and then apply different learning mechanisms: a conformist bias where the song is consistent within the local population or social network, cultural drift or a rare-form bias in highly variable songs or segments, and demonstrator biases for song features that are characteristic of dominant males. Conformist biases may not apply when a song trait varies over a wide range along a continuum, coming into play only when the same range is separated into categories. Well-designed studies will be necessary to establish whether wild songbirds define song categories based on distributions of acoustic features in the song population and whether such categories can be the basis of conditional learning rules.

Integration of laboratory and field studies has long been a valuable aspect of songbird learning studies. Field observations give rise to hypotheses that are tested in laboratory investigations, which in turn contribute to the design of field playback studies. Carefully designed playback stimuli are especially important and such studies must also be timely if they are to illuminate the mechanisms of ongoing cultural evolution. Long-term field studies tracking demography, social networks, reproductive success, and status – as well as song transmission – will also be critical for understanding the relative roles of different learning biases. Large-scale field experiments, such as the seeding of novel variant forms in a population, will provide an additional tool for understanding the roles of different mechanisms in song transmission and the cultural evolution of song in wild populations.

## Author Contributions

The author confirms being the sole contributor of this work and has approved it for publication.

## Conflict of Interest

The author declares that the research was conducted in the absence of any commercial or financial relationships that could be construed as a potential conflict of interest.

## Footnotes

^1^This review focuses upon the songs of migratory species that breed in the temperate zones of the Northern Hemisphere. In most of these species, males sing and females do not (this statement is also true of zebra finches, which in the wild are neither migratory nor inhabitants of the Northern Hemisphere). However, females of many species do sing, and song learning in both sexes is probably the ancestral form of this behavioral trait (Odom et al., 2014).
